# Multiplex Real-Time PCR Assays that Measure the Abundance of Extremely Rare Mutations Associated with Cancer

**DOI:** 10.1371/journal.pone.0156546

**Published:** 2016-05-31

**Authors:** Diana Y. Vargas, Fred Russell Kramer, Sanjay Tyagi, Salvatore A. E. Marras

**Affiliations:** 1 Public Health Research Institute, Rutgers University, Newark, New Jersey, United States of America; 2 Department of Microbiology, Biochemistry and Molecular Genetics, New Jersey Medical School, Rutgers University, Newark, New Jersey, United States of America; 3 Department of Medicine, New Jersey Medical School, Rutgers University, Newark, New Jersey, United States of America; University of Navarra, SPAIN

## Abstract

We describe the use of “SuperSelective” primers that enable the detection and quantitation of somatic mutations whose presence relates to cancer diagnosis, prognosis, and therapy, in real-time PCR assays that can potentially analyze rare DNA fragments present in blood samples (liquid biopsies). The design of these deoxyribonucleotide primers incorporates both a relatively long “5' anchor sequence” that hybridizes strongly to target DNA fragments, and a very short, physically and functionally separate, “3' foot sequence” that is perfectly complementary to the mutant target sequence, but mismatches the wild-type sequence. As few as ten mutant fragments can reliably be detected in the presence of 1,000,000 wild-type fragments, even when the difference between the mutant and the wild type is only a single nucleotide polymorphism. Multiplex PCR assays employing a set of SuperSelective primers, and a corresponding set of differently colored molecular beacon probes, can be used in situations where the different mutations, though occurring in different cells, are located in the same codon. These non-symmetric real-time multiplex PCR assays contain limited concentrations of each SuperSelective primer, thereby enabling the simultaneous determination of each mutation’s abundance by comparing its threshold value to the threshold value of a reference gene present in the sample.

## Introduction

It has been a long-sought medical goal to be able to detect at a very early stage extremely rare mutations whose presence in a clinical sample is useful for diagnosing cancer, determining prognosis, and indicating the choice of effective therapy [1]. The detection and quantitative assessment of relevant somatic mutations has multiple uses, including: (i) the detection of cancer at a treatable stage in patients who inherit genes that make cancer more likely; (ii) the detection of mutations in benign cancer cells that indicate that they may now metastasize; (iii) measurement of the abundance of cancer cells during treatment; and (iv) the determination as to whether drug-resistant cancer cells have arisen during treatment, so that therapy can be adjusted. A further goal is to develop methods that enable multiplex assays that can simultaneously measure the abundance of different rare mutations. If such assays were to become available, cancer could potentially be converted from an often-fatal disease to a chronic condition that can be managed by frequent testing combined with individualized therapeutic adjustments.

Spurring these efforts on is the realization that cancer cells, no matter where in the body they are located, divide frequently, undergo apoptosis and necrosis, and as a consequence, genomic DNA fragments from those cancer cells are present in each patient's blood plasma [2]. This realization has opened up the possibility that the presence of rare mutations indicative of cancer diagnosis, prognosis, and treatment can be detected and quantitated at a very early stage, by performing "liquid biopsies," utilizing DNA isolated from plasma [3–5]. The challenge facing assay designers is to find a means of selectively detecting and quantitating these rare mutant sequence fragments in plasma DNA, despite the presence of abundant wild-type sequence fragments originating from normal cells throughout the body, and despite the fact that different relevant mutations, though originating in different cells, often occur in the same or adjacent codons. The success of "next-generation" sequencing for the detection of rare mutant sequence fragments in plasma DNA [6–8], though complex and costly, has illustrated the value of this approach.

Molecular diagnostic assays based on the exponential amplification of nucleic acid target sequences, such as polymerase chain reactions, are inexpensive and sufficiently sensitive to generate signals from as little as a single template molecule. The challenge is to design these assays to be extraordinarily selective, so that they enable the exponential amplification of mutant DNA fragments, while simultaneously suppressing the generation of amplicons from far more abundant wild-type DNA fragments, even when the only difference between the mutant sequence and the wild-type sequence is a single-nucleotide polymorphism.

Promising approaches utilize amplification primers that are designed to be highly selective. For example, the nucleotide sequences of amplification refractory mutation system (ARMS) primers [9] are perfectly complementary to mutant target sequences, but contain an "interrogating nucleotide" at their 3' end that is not complementary to the corresponding nucleotide in wild-type target sequences. The resulting mismatched wild-type hybrids are much less likely to enable the synthesis of amplicons, because DNA polymerases require a 3'-terminal base pair to initiate synthesis. Here, the selective mechanism is enzymatic. On the other hand, dual priming oligonucleotide (DPO) primers [10], MyT primers [11], hairpin primers [12, 13], and PASS primers [14], utilize a different mechanism. They too possess a priming sequence that is perfectly complementary to a mutant target, but contain an internal interrogating nucleotide that mismatches the corresponding wild-type sequence. The length of their priming sequence is chosen so that, under annealing conditions, perfectly complementary mutant hybrids are likely to form, and are therefore likely to lead to the generation of amplicons, while mismatched wild-type hybrids are much less likely to form, and are therefore much less likely to lead to the generation of amplicons. Alternative approaches, involving PCR-clamping [15] or the use of hairpin oligonucleotide blockers [16], utilize a mixture that contains both conventional DNA primers that bind to mutant sequences, and "anti-primers" that are designed to bind selectively to wild-type sequences, thereby preventing the initiation of wild-type amplicon synthesis. However, all of these approaches, though generally applicable for the detection of mutant sequences, are either not sufficiently sensitive to detect extremely rare mutants [12–15], not compatible with real-time PCR due to the presence of unnatural nucleotides in their sequence [10], or have not been shown to enable quantitative determinations in multiplex real-time PCR assays when different target mutations occur in the same codon [9, 11, 16].

This report describes experiments that explore the design and function of “SuperSelective” PCR primers that enable the quantitation of rare mutant targets. Significantly, once these primers initiate synthesis on mutant DNA fragments, the resulting amplicons are exponentially amplified with high efficiency in subsequent thermal cycles, and the real-time data provide a conventional means of assessing the abundance of the mutant templates in the original sample. In addition, this report describes particular designs for the SuperSelective primers, and modifications to the PCR format, that enable multiplex real-time PCR assays to be carried out that measure the abundance of different mutant target sequences relative to the abundance of a reference wild-type sequence present in each sample.

## Materials and Methods

### Primers and molecular beacons

SuperSelective primer sequences were examined with the aid of the Mfold web server [17] and the OligoAnalyzer computer program (Integrated DNA Technologies, Coralville, IA) to ensure that under assay conditions they are unlikely to form internal hairpin structures, and are unlikely to form self-dimers or heterodimers with the conventional reverse primers. The primers were purchased from Integrated DNA Technologies; and the differently colored molecular beacon probes for detecting the amplicons were purchased from Biosearch Technologies (Petaluma, CA).

### DNA templates

Plasmids containing *EGFR* sequences (either the L858 mutant sequence or the wild-type sequence) were prepared by inserting a 115-base pair gene fragment into a pGEM^®^-11Zf(+) vector (Promega, Madison, WI). The sequences of these plasmids were confirmed by sequence analysis. Human genomic DNA encoding the *EGFR* L858R mutation was isolated from cell line H1975 (CRL-5908, American Type Culture Collection, Manassas, VA) and human genomic DNA containing the corresponding wild-type sequence was purchased from Coriell Cell Repositories (Camden, NJ). The mutant and wild-type *EGFR* plasmids, and the human genomic DNAs, were digested by incubation with restriction endonuclease Mse I (New England Biolabs, Ipswich, MA). The 20-μl digestion mixtures contained 4 μg of DNA, 10 units of Mse I, 50 mM NaCl, 10 mM MgCl_2_, 100 μg/mL bovine serum albumin, and 10 mM Tris-HCl (pH 7.9). These reactions were incubated for 120 min at 37°C, followed by incubation for 20 min at 65°C to inactivate the endonuclease.

Plasmids containing *BRAF* sequences (either the V600E mutant sequence, the V600R mutant sequence, or the wild-type sequence) were purchased from Integrated DNA Technologies, and were prepared by inserting a 200-base-pair gene fragment into pIDTSmart Amp vectors. The *BRAF* plasmids were digested by incubation with restriction endonuclease Sca I (New England Biolabs). The 20-μl digestion mixtures contained 4 μg of DNA, 10 units of Sca I, 100 mM NaCl, 10 mM MgCl_2_, 1 mM dithiothreitol, and 50 mM Tris-HCl (pH 7.9). These reactions were incubated for 120 min at 37°C, followed by incubation for 20 min at 80°C to inactivate the endonuclease.

### PCR assays

Monoplex real-time polymerase chain reactions were performed in 30-μl volumes containing 50 mM KCl, 3 mM MgCl_2_, 10 mM Tris-HCl (pH 8.0), 250 μM dATP, 250 μM dCTP, 250 μM dGTP, 250 μM dTTP, 1.5 units of AmpliTaq Gold DNA polymerase (ThermoFisher Scientific, Waltham, MA), 120 nM of each primer, and 1x SYBR^®^ Green (ThermoFisher Scientific) for monitoring amplicon abundance during the chain elongation stage of each thermal cycle.

Duplex real-time polymerase chain reactions were performed in 30-μl volumes containing 50 mM KCl, 2.5 mM MgCl_2_, 10 mM Tris-HCl (pH 8.0), 250 μM dATP, 250 μM dCTP, 250 μM dGTP, 250 μM dTTP, 1.5 units of Platinum Taq DNA polymerase (ThermoFisher Scientific), either 500 nM (symmetric PCR) or 60 nM (non-symmetric PCR) of each *BRAF* SuperSelective primer, 1,000 nM of the conventional *BRAF* common reverse primer, and 300 nM of each molecular beacon for monitoring the abundance of each type of amplicon during the annealing stage of each thermal cycle.

Triplex real-time polymerase chain reactions contained the same reagents as the duplex reactions, except that they contained 60 nM of each *BRAF* SuperSelective primer, 60 nM of the *EGFR* SuperSelective primer, 1,000 nM of the conventional *BRAF* common reverse primer, and 500 nM of the conventional *EGFR* reverse primer. We utilized *EGFR* wild-type plasmids as the reference gene target in these model triplex assays, since they were available in our laboratory.

All amplifications were carried out in 200-μl white polypropylene PCR tubes (USA Scientific, Ocala, FL) in an IQ5 spectrofluorometric thermal cycler (Bio-Rad Laboratories, Hercules, CA). The reaction mixtures were incubated for 10 min at 95°C to activate the AmpliTaq Gold DNA polymerase (monoplex reactions) or were incubated for 2 min at 95°C to activate the Platinum Taq DNA polymerase (multiplex reactions), followed by 55 or 60 cycles consisting of 95°C denaturation for 20 sec, 60°C annealing for 20 sec, and 72°C chain elongation for 20 sec.

## Results

SuperSelective primers are oligodeoxyribonucleotides whose function in a PCR assay has been divided into two parts [10, 14, 18]. The function of efficiently binding to a gene of interest is assigned to a relatively long 5' sequence segment (which we call the “anchor”), and the function of selectively binding to a nearby subsequence within that gene that contains the mutation of interest, and then initiating the synthesis of an amplicon, is assigned to a separate, short 3' sequence segment (which we call the “foot”). The foot contains an “interrogating nucleotide” that is complementary to the corresponding nucleotide in the mutant target sequence, but mismatches the corresponding nucleotide in the wild-type target sequence. In SuperSelective primers, the anchor is separated from the foot by an additional, relatively long, deoxyribonucleotide sequence segment (which we call the “bridge”). The bridge is chosen so as to insure that it does not form secondary structures and is not complementary to the "intervening sequence" in the template molecule that joins the anchor target sequence to the foot target sequence. Consequently, when the primer is hybridized to a template molecule, the bridge sequence in the primer and the intervening sequence in the template form a single-stranded "bubble" that functionally separates the efficient formation of the anchor hybrid from the formation of the foot hybrid. The resulting primers are bifunctional: under annealing conditions, the long 5' anchor sequence enables the primer to bind efficiently and selectively to the genomic region of interest present in the target DNA fragments, while the short 3' foot sequence (which possesses the interrogating nucleotide), because it is tethered to the anchor sequence by the bridge sequence, is able to form a weak (perfectly complementary) hybrid with the mutant target sequence; yet due to its short length, the foot is unlikely to form a considerably weaker (mismatched) hybrid with the corresponding wild-type sequence.

**Fig 1:**
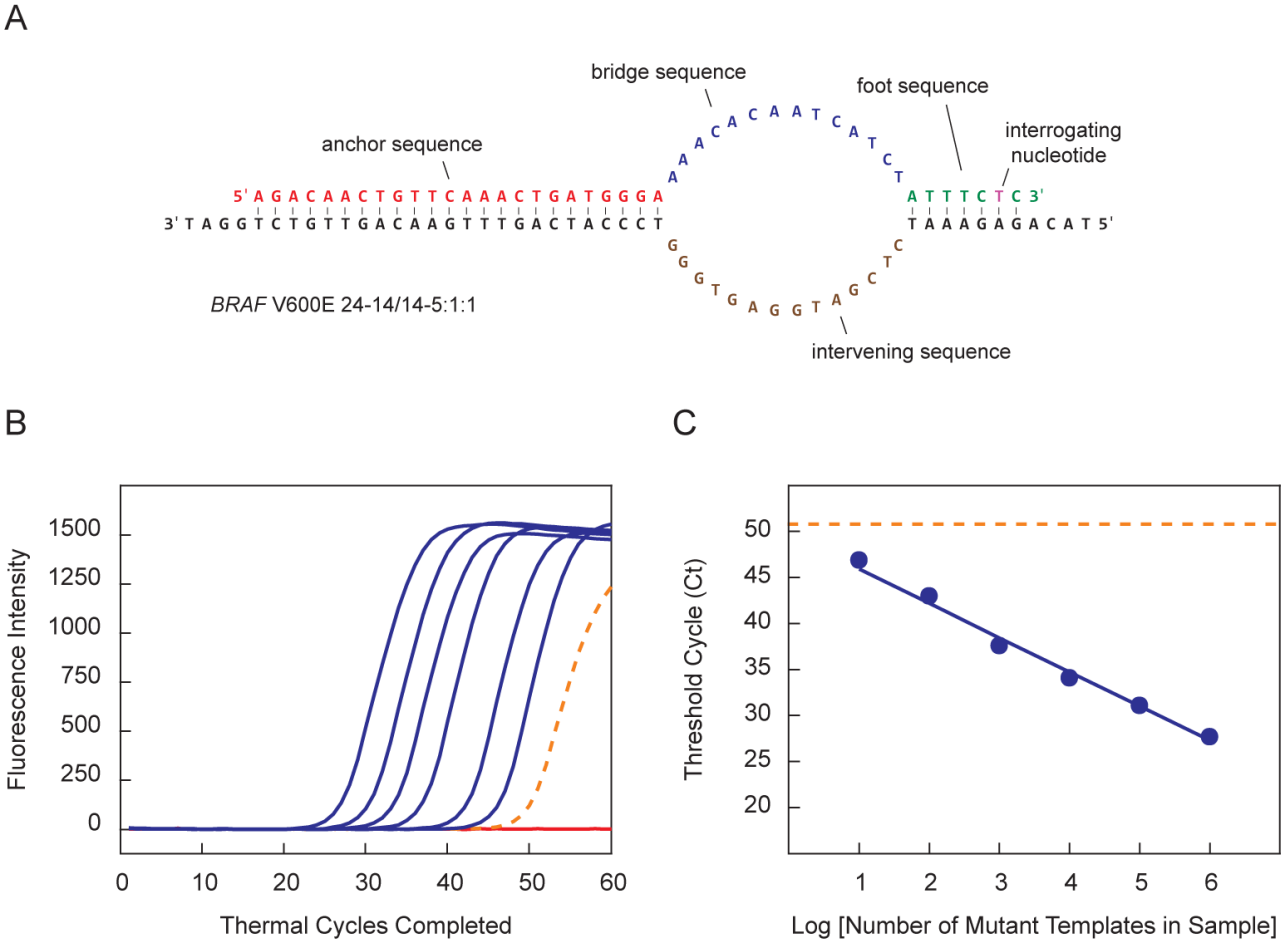
Structure of a SuperSelective primer for the detection of *BRAF* V600E mutant sequences in the presence of abundant *BRAF* wild-type sequences, and a demonstration of its use in monoplex real-time PCR assays. (**A**) SuperSelective primer *BRAF* V600E 24-14/14-5:1:1 contains a long 5'-anchor sequence that binds strongly to template strands, a short 3'-foot sequence that includes an interrogating nucleotide that is perfectly complementary to the corresponding nucleotide in a mutant template (but mismatches the corresponding nucleotide in a wild-type template), and a bridge sequence that links the anchor sequence to the foot sequence, and that is chosen to not be complementary to the corresponding intervening sequence in the template strand, thereby forming a single-stranded bubble that separates the function of the anchor from the function of the foot. (**B**) Real-time PCR assays employing SuperSelective primer *BRAF* V600E 24-14/14-5:1:1. Six reactions initiated with 10^6^ *BRAF* wild-type templates plus different quantities of mutant templates (10^1^, 10^2^, 10^3^, 10^4^, 10^5^, and 10^6^) are plotted in blue; a reaction initiated with only 10^6^ wild-type templates is plotted with a dotted orange line; and a control reaction containing no template DNA is plotted in red. (**C**) The threshold cycle measured for each reaction that contained mutant templates is plotted as a function of the logarithm of the number of mutant templates initially present in each reaction. The dotted orange line indicates the threshold cycle of the reaction containing only wild-type templates.

Fig 1A shows an example of a SuperSelective primer bound to its mutant target sequence. This particular primer was designed to selectively amplify DNA fragments containing the *BRAF* V600E single-nucleotide polymorphism, whose presence in cells predisposes patients to hereditary nonpolyposis colorectal cancer [19, 20]. We refer to this primer as “*BRAF* V600E 24-14/14-5:1:1”, which indicates that the anchor sequence is 24 nucleotides long, the bridge sequence is 14 nucleotides long (across from an intervening sequence in the template that is also 14 nucleotides long), and the foot sequence is 7 nucleotides long, with the interrogating nucleotide located at the penultimate position from the primer’s 3' end.

### Real-time PCR assays containing SuperSelective primers

Real-time PCR assays were carried out, whose only difference from a conventional real-time PCR assay was the replacement of the conventional forward primer with the *BRAF* V600E SuperSelective forward primer shown in Fig 1A. These monoplex assays contained a conventional reverse primer that was present at the same concentration as the SuperSelective primer. Eight 30-μL PCR assays were prepared. Seven of the reactions contained DNA fragments from 10^6^ plasmids possessing the *BRAF* wild-type sequence and DNA fragments from either 10^6^, 10^5^, 10^4^, 10^3^, 10^2^, 10^1^, or 0 plasmids possessing the single-nucleotide polymorphism in the *BRAF* V600E mutant sequence. An eighth reaction that contained no DNA fragments served as a control. All of the reactions contained the intercalating DNA dye, SYBR^®^ Green, whose fluorescence intensity during the chain elongation phase of each thermal cycle reflects the number of amplicons synthesized.

Fig 1B shows the results of this experiment. The control reaction that contained no template DNA did not produce any false amplicons, such as primer-dimers, despite the longer length of the SuperSelective primers. The reaction containing 1,000,000 wild-type templates and no mutant templates was suppressed to such an extent that it did not produce a significant number of amplicons until about 50 cycles of amplification had been carried out. Yet, the six reactions initiated with 1,000,000 wild-type templates and different numbers of mutant templates took significantly fewer cycles of amplification to generate a significant number of amplicons. When the threshold cycle (Ct) of each of these six reactions was plotted against the logarithm of the number of mutant templates in each reaction, the result was a straight line (Fig 1C). This inverse linear relationship between the logarithm of the number of mutant targets originally present in a sample and the Ct value observed for that sample is the hallmark of quantitative exponential amplification assays [21, 22]. Significantly, the Ct value from the sample containing 10 mutant templates in the presence of 1,000,000 wild-type templates was readily distinguishable from the Ct value generated by the sample containing only 1,000,000 wild-type templates (shown in the figure as a dotted orange line).

**Fig 2:**
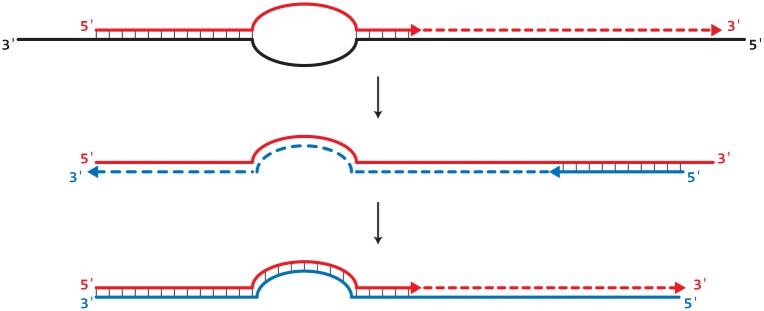
Principle of operation of SuperSelective primers. The selective step occurs only when a SuperSelective primer hybridizes to a DNA (-) template fragment present in the sample. Due to the small size of the foot sequence, the probability of initiation of a (+) amplicon is significantly greater if the target sequence of the foot in the (-) template fragment is a completely complementary mutant sequence, than if the target sequence of the foot in the (-) template fragment is a mismatched wild-type sequence. If (+) amplicon synthesis does occur, then the resulting (+) amplicon serves as a template for a conventional reverse primer, and is efficiently copied during the next thermal cycle, generating a (-) amplicon in which the complement of the unique bridge sequence that was present in SuperSelective primer is substituted for the intervening sequence that was present in the original (-) template fragment. As a result, in subsequent thermal cycles, the entire SuperSelective primer sequence is complementary to the (-) amplicon strands, and exponential amplification occurs efficiently, and can be followed in real-time.

In these assays, the selective step occurs when a SuperSelective primer is bound to a DNA (-) strand template that is present in the original sample being analyzed. Once the foot sequence of a SuperSelective primer initiates the synthesis of an amplicon, the entire sequence of the SuperSelective primer (including the “artificial” bridge sequence) is incorporated into that (+) amplicon. In subsequent thermal cycles, the resulting amplicons are amplified efficiently in the normal manner, with the entire SuperSelective primer sequence serving as a long conventional primer that is completely complementary to the (-) amplicons, which include the complement of the primer’s bridge sequence in place of the intervening sequence that was present in the original template (Fig 2).

### Optimization of the design of SuperSelective primers

The three parts of a SuperSelective primer serve different functions. The 5' anchor sequence is designed so that it is long enough and strong enough so that at the annealing temperature of the PCR assay it binds to the target sequence, irrespective of whether or not the target sequence is mutant or wild type. The short 3' foot sequence, on the other hand, is designed to be able to bind to the completely complementary mutant target sequence at the annealing temperature of the PCR assay, but to not bind to the mismatched wild-type sequence under the same conditions. The role of the bubble, formed by the bridge sequence in the primer and the intervening sequence in the template, is two-fold: (i) by separating the anchor sequence from the foot sequence, the presence of the single-stranded bubble causes the formation of the weak foot hybrid to occur independently from the formation of the strong anchor hybrid; and (ii) by tethering the foot sequence in the primer to the potential target sequence in the template, the probability that the short foot sequence (6, 7, or 8 nucleotides in length) will actually form a hybrid is markedly increased. A free-floating sequence the size of the foot would virtually never form a hybrid with a target sequence under PCR annealing conditions. In order to better understand the role played by the different sequence segments of SuperSelective primers, and in order to understand how best to design these primers so that they are highly selective for any given target sequence, we carried out a series of model assays that explored the optimal design of the foot sequence, and the optimal design of the bubble.

**Fig 3:**
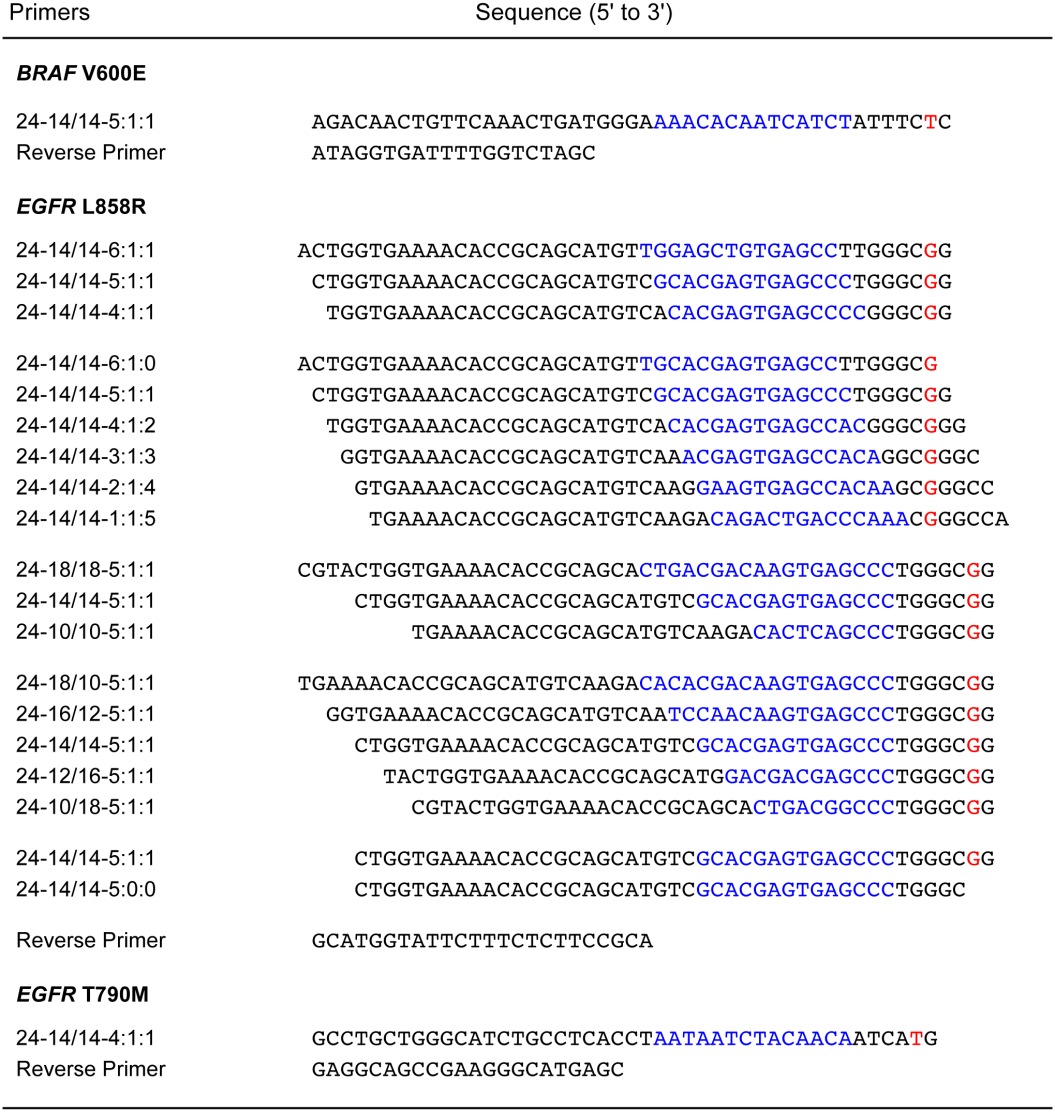
Primers utilized in monoplex PCR assays. The bridge sequence within each SuperSelective primer is shown in blue, and the interrogating nucleotide in each foot sequence is shown in red. The *EGFR* L858R primers are arranged into groups that reflect their use in comparative experiments. The *BRAF* V600E target sequence is 69 base pairs long; the *EGFR* L858R target sequences are between 79 and 87 base pairs long, depending on the SuperSelective primer that is used; and the *EGFR* T790M target sequence is 68 base pairs long.

The wild-type sequence in which the *BRAF* V600E mutation occurs is A-T rich (3'-TAAAGTG-5'), thereby ensuring that the weak mismatched hybrid that it forms with the foot of the *BRAF* V600E 24-14/14-5:1:1 SuperSelective primer is very unlikely to form under the annealing conditions of the PCR assay [23], leading to the significant suppression of wild-type amplicon synthesis (Fig 1). However, when we carried out similar assays with a SuperSelective primer designed to detect the single-nucleotide polymorphism in the *EGFR* L858R mutant sequence, whose presence in non-small-cell lung cancer predicts resistance to tyrosine kinase inhibitors [24, 25], we found that its presence in a G-C rich wild-type sequence (3'-ACCCGAC-5') resulted in less suppression of wild-type amplicon synthesis. We therefore carried out a series of experiments with different *EGFR* L858R SuperSelective primer designs (Fig 3) to optimize discrimination between the mutant sequence and its almost identical wild-type sequence.

### Effect of varying the length of the foot sequence

**Fig 4:**
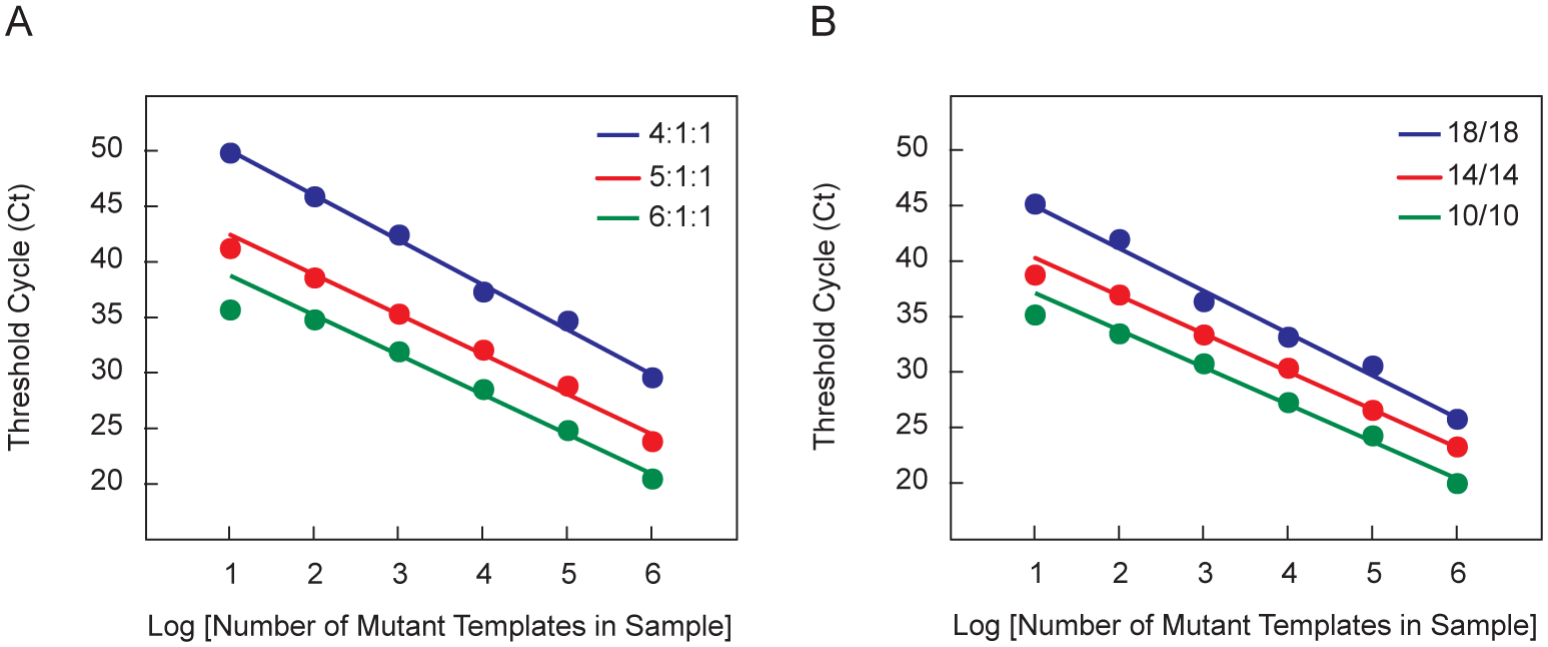
SuperSelective primer elements that affect selectivity. The linearity of the relationship between the threshold cycle and the logarithm of the initial number of mutant templates present in reactions containing 10^6^ wild-type templates and different amounts of mutant template was determined for PCR assays initiated with different SuperSelective primer designs. (**A**) PCR reactions initiated with *EGFR* L858R SuperSelective primers possessing foot sequences of different length. (**B**) PCR reactions initiated with *EGFR* L858R SuperSelective primers that form symmetrical bubbles of different circumference.

We explored the effect of shortening the length of the SuperSelective primer's foot sequence to overcome the higher probability that the foot will form a hybrid with the G-C rich sequence present in the *EGFR* wild-type target. We carried out three sets of assays, each set utilizing a SuperSelective primer whose foot sequence was 6, 7, or 8 nucleotides in length. In all other respects, the design of the primers was the same: (i) the interrogating nucleotide was located at the penultimate position at the 3' end of each foot; (ii) the anchor sequence was 24 nucleotides long; and (iii) the bridge sequence and the intervening sequence were each 14 nucleotides long. The sequences of these three primers (*EGFR* L858R 24-14/14-6:1:1; *EGFR* L858R 24-14/14-5:1:1; and *EGFR* L858R 24-14/14-4:1:1) are shown in Fig 3. Each set of PCR assays was initiated with different quantities of mutant template (10^6^, 10^5^, 10^4^, 10^3^, 10^2^, and 10^1^ copies) in the presence of 10^6^ copies of wild-type template. The resulting threshold values are plotted as a function of the logarithm of the number of mutant templates initially present (Fig 4A).

The Ct values obtained with the primer possessing the shortest foot length (six nucleotides, 4:1:1) all fall on a straight line, demonstrating that even though the *EGFR* target sequence is G-C rich, as few as 10 mutant templates could be quantitated without interference from the 1,000,000 wild-type templates that were present. Primers possessing longer foot lengths (seven nucleotides, 5:1:1; or eight nucleotides, 6:1:1), however, led to a divergence from linearity when the mutant targets are most rare, due to inadequate suppression of amplicon synthesis on the abundant wild-type templates. These results demonstrate that shorter foot lengths, though lowering the equilibrium abundance of foot hybrids, resulting in longer delays before the threshold cycle is achieved, lead to enhanced selectivity. From a thermodynamic standpoint, the improved selectivity at shorter foot lengths is due to the higher ratio of the equilibrium abundance of perfectly complementary mutant foot hybrids compared to the equilibrium abundance of mismatched wild-type foot hybrids.

### Effect of varying the location of the interrogating nucleotide

We also investigated the effect of varying the location of the interrogating nucleotide in the foot sequence on the primer's ability to discriminate mutant templates from wild-type templates. We carried out a series of PCR assays in which six different SuperSelective primers were utilized, each possessing an interrogating nucleotide at a different position within a 7-nucleotide-long foot sequence. The lengths of the anchor sequence, bridge sequence, and intervening sequence were maintained at 24, 14, and 14 nucleotides, respectively. The sequences of these six *EGFR* L858R primers are shown in Fig 3. Two reactions were carried out with each primer, one initiated with 1,000,000 copies of mutant template, and one initiated with 1,000,000 copies of wild-type template. The threshold cycles that were observed are listed in Table 1. The results show that the window of discrimination (ΔCt) between the threshold cycle for the mutant and the threshold cycle for the wild type is widest when the location of the interrogating nucleotide is closest to the 3' end of the primer. Because there is only a small difference in ΔCt between the primer whose interrogating nucleotide was located at the 3' end of the foot (ΔCt = 18.8), and the primer whose interrogating nucleotide was located at the penultimate position from the 3' end of the foot (ΔCT = 18.2), we conclude that placement of the interrogating nucleotide at either position works well. Since the interrogating nucleotide at the penultimate position from the 3' end of the foot does not form a base pair with the corresponding nucleotide in the wild-type sequence, under PCR annealing conditions, the 3'-terminal nucleotide of the foot is very unlikely to form a base pair with its corresponding complementary nucleotide in the wild-type sequence.

**Table 1:** Threshold cycles observed for PCR assays containing SuperSelective *EGFR* L858R primers whose interrogating nucleotide is located at different positions within the foot sequence.

	Threshold Cycle (Ct)		
Primer	10^6^ Mutant Templates	10^6^ Wild-type Templates	ΔCt
24-14/14-6:1:0	24.3	43.1	18.8
24-14/14-5:1:1	22.9	41.1	18.2
24-14/14-4:1:2	21.2	36.1	14.9
24-14/14-3:1:3	23.0	35.2	12.2
24-14/14-2:1:4	23.1	33.2	10.1
24-14/14-1:1:5	21.1	30.4	9.3

### Effect of varying the circumference of the bubble

We also investigated the effect of varying the circumference of the single-stranded bubble that functionally separates the anchor hybrid from the foot hybrid (see Fig 1). We carried out three sets of PCR assays that were identical to the experiment whose results are shown in Fig 4A, except that three different SuperSelective primers were utilized, each forming symmetrical bubbles of different circumference when hybridized to its template. Each of these three primers possessed the same 7-nucleotide-long foot sequence, with the interrogating nucleotide located at the penultimate position from its 3' end, and the length of the anchor sequence in each of the primers was maintained at 24 nucleotides. However, the bridge sequence in each primer was chosen to be 10, 14, or 18 nucleotides in length, and the identity of the anchor sequence in each primer was chosen so as to create an intervening sequence in the template that was the same length as the bridge sequence. Consequently, the circumference of the bubble formed by each of these primers when fully hybridized to a template (consisting of the bridge sequence, the intervening sequence, the two nucleotides on the end of the anchor hybrid, and the two nucleotides on the end of the foot hybrid) was 24, 32, or 40 nucleotides in length. The sequences of these primers (*EGFR* L858R 24-10/10-5:1:1; *EGFR* L858R 24-14/14-5:1:1; and *EGFR* L858R 24-18/18-5:1:1) are shown in Fig 3. Each set of PCR assays was initiated with different quantities of mutant template (10^6^, 10^5^, 10^4^, 10^3^, 10^2^, and 10^1^ copies) in the presence of 10^6^ copies of wild-type template. The resulting threshold values are plotted as a function of the logarithm of the number of mutant templates initially present (Fig 4B).

The Ct values obtained with the primer forming the largest bubble (18/18) all fall on a straight line, demonstrating that even though the *EGFR* target sequence is G-C rich, as few as 10 mutant templates could be quantitated without interference from the 1,000,000 wild-type templates that were present. Primers possessing smaller bubbles (14/14 and 10/10), however, lead to a divergence from linearity when there are only 10 mutant targets present, apparently due to inadequate suppression of amplicon synthesis on the abundant wild-type templates. The superior linearity of the data obtained with the primer that forms the largest bubble (resulting in longer delays before the threshold cycle is achieved), indicates that reducing the equilibrium abundance of both the mutant hybrids and the wild-type hybrids (as a consequence of the greater entropic freedom of the foot relative to its target) enhances the selectivity of the assay.

### Effect of varying the symmetry of the bubble

We also investigated the effect of altering the symmetry of the bubble on the primer's ability to discriminate mutant templates from wild-type templates. We carried out a series of PCR assays in which five different SuperSelective primers were utilized, each of which formed bubbles that had the same circumference, but each of which possessed bridge sequences of differing length. In these primers, the identity of the anchor sequence was chosen so as to create an intervening sequence in the template whose length, in combination with the length of the bridge sequence in the primer, resulted in a bubble whose circumference was 32 nucleotides. The sequences of these five *EGFR* L858R primers (24-18/10-5:1:1; 24-16/12-5:1:1; 24-14/14-5:1:1; 24-12/16-5:1:1; and 24-10/18-5:1:1) are shown in Fig 3. Two reactions were carried out for each of the primers, one initiated with 1,000,000 copies of mutant template, and one initiated with 1,000,000 copies of wild-type template. The threshold cycles that were observed are listed in Table 2. The results show that the window of discrimination (ΔCt) between the threshold cycle for the mutant and the threshold cycle for the wild type is similar for all of the primers, irrespective of whether the bubble is symmetric or asymmetric, although the symmetrical bubble did provide the greatest selectivity. What mainly matters is the circumference of the bubble, which determines the equilibrium probability of a foot sequence encountering a target sequence.

**Table 2:** Threshold cycles observed for PCR assays containing SuperSelective *EGFR* L858R primers that form bubbles with varying symmetries.

	Threshold Cycle (Ct)		
Primer	10^6^ Mutant Templates	10^6^ Wild-type Templates	ΔCt
24-18/10-5:1:1	22.8	39.3	16.5
24-16/12-5:1:1	22.1	38.2	16.1
24-14/14-5:1:1	22.9	41.1	18.2
24-12/16-5:1:1	22.5	38.4	15.9
24-10/18-5:1:1	22.1	39.5	17.4

### Selective amplification of mutant sequences in samples containing human genomic DNA

The assays described above were carried out with DNA fragments obtained from plasmids that were digested with restriction endonucleases in order to mimic the target sequences that occur in plasma samples. Clinical plasma samples, however, contain DNA fragments from the entire human genome. Although the number of DNA fragments in a clinical sample is highly variable from person-to-person, and from time-to-time in a given person, there are usually no more than 10,000 wild-type template fragments related to each target mutation in samples obtained from 10 mL of blood [26]. We have discovered that in designing assays for use with plasma samples, the SuperSelective primers can be somewhat less selective than those used in the model assays described above (i.e., they can form somewhat smaller bubbles, and they can possess slightly longer foot sequences), and as a consequence, the probability of initiating the synthesis of an amplicon is higher and the resulting amplification curves will have earlier Ct values.

**Fig 5:**
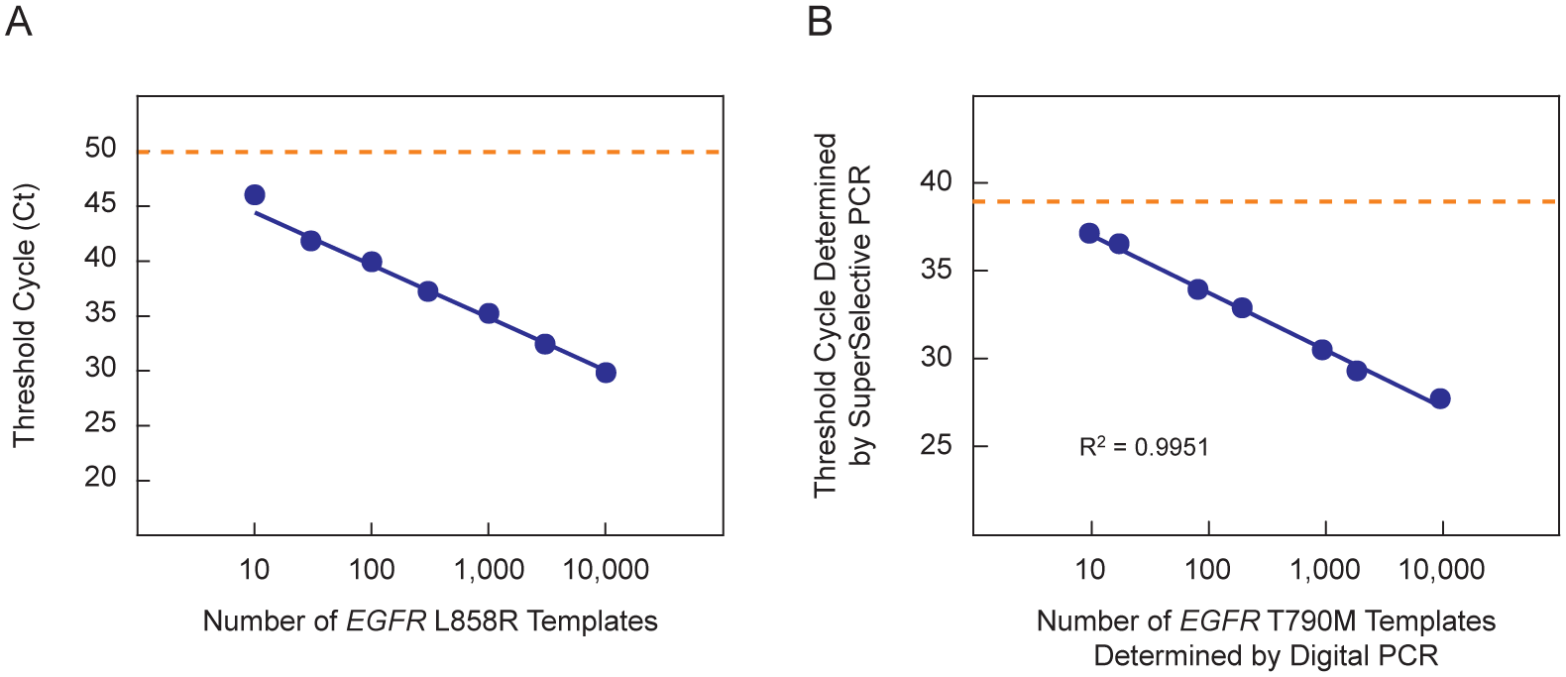
Assays of samples containing human genomic DNA. (**A**) To mimic assays initiated with DNA fragments isolated from blood plasma, we utilized SuperSelective primer *EGFR* L858R 24-14/14-5:1:1 in a set of eight PCR assays that were initiated with different quantities of digested human genomic DNA encoding the *EGFR* L858R mutation (DNA from 0; 10; 30; 100; 300; 1,000; 3,000; or 10,000 cells) in the presence of digested human genomic DNA from 10,000 wild-type human cells. The threshold cycle measured for each reaction that contained mutant templates is plotted as a function of the logarithm of the number of mutant templates initially present in each reaction. The dotted orange line indicates the threshold cycle of the reaction containing only wild-type templates. (**B**) Comparison of the number of *EGFR* T790M mutant target molecules present in human genomic DNA samples (as determined by droplet digital PCR) to the Ct values obtained for the same samples in real-time PCR assays that utilize SuperSelective primer *EGFR* T790M 24-14/14-4:1:1. The dotted orange line indicates the threshold cycle of a reaction containing only wild-type templates.

To mimic assays initiated with DNA fragments isolated from blood plasma, we therefore utilized the *EGFR* L858R 24-14/14-5:1:1 primer in a set of eight PCR assays that were initiated with samples that contained different quantities of restriction enzyme-digested human genomic DNA isolated from cell line H1975, which harbors the *EGFR* L858R mutation (DNA from 0; 10; 30; 100; 300; 1,000; 3,000; or 10,000 cells) in the presence of restriction-enzyme digested human genomic DNA isolated from 10,000 cells that contain wild-type *EGFR* genes. The results are shown in Fig 5A. The linearity of the plot of the Ct values as a function of the logarithm of the number of mutant fragments initially present in each reaction confirms the specificity of assays that utilize SuperSelective primers when the samples contain genomic DNA fragments. Moreover, the high Ct value of the reaction initiated with only wild-type DNA (shown in the figure as a dotted orange line) confirms that the primer’s selectivity is unaffected by the presence of genomic DNA fragments. The significance of this experiment is that SuperSelective primers, by virtue of their bifunctionality (both the anchor and the foot must form hybrids for amplicons to be synthesized) are so specific that they are unaffected by the presence of DNA fragments from the rest of the human genome.

### Comparison of digital PCR to real-time SuperSelective PCR using human genomic DNA

To confirm that the Ct values observed in real-time PCR assays that utilize SuperSelective primers reflect the actual number of target molecules present in a sample, we compared those Ct values to the number of target molecules determined for the same samples by our colleagues at MolecularMD (Portland, OR) with a Bio-Rad droplet digital PCR system. We used samples (prepared by Molecular MD) that contained human genomic DNA isolated from approximately 6,000 wild-type cells and different amounts of human genomic DNA isolated from cells possessing the *EGFR* T790M single-nucleotide polymorphism, which is another mutation whose presence in non-small-cell lung cancer predicts resistance to tyrosine kinase inhibitors [27]. The sequence of SuperSelective primer *EGFR* T790M 24-14/14-4:1:1 is shown in Fig 3. The results are shown in Fig 5B. There was a strong correlation between the number of target molecules present as determined by digital PCR and the Ct values determined by real-time PCR using SuperSelective primers (r^2^ = 0.9951). Moreover, the Ct value for a sample containing only DNA from wild-type cells (shown in the figure as a dotted orange line) occurred later than the Ct value of the sample containing DNA from 10 mutant cells in the presence of DNA from wild-type cells.

### Targeting different mutations located in the same codon

When rare mutant sequences occur in separate genes, multiplex real-time PCR assays can be designed to quantitate the amount of each mutant gene in the same sample, because each SuperSelective primer will have a different anchor sequence, and the amplicons generated from each mutant gene will possess a unique sequence that is easily distinguished from the sequences of amplicons generated from the other mutant genes; and these amplicons can be distinguished from each other through the use of differently colored fluorescent probes, such as TaqMan probes [28, 29], molecular beacon probes [30, 31], or strand displacement probes [32]. However, rare mutant sequences that are relevant to cancer diagnosis, prognosis, and treatment, although usually occurring in different cells, are often located within the same or adjacent codons of the same gene. For example, four key mutations are located within codon 600 of the *BRAF* gene [33], and seven key mutations are located within adjacent codons 12 and 13 of the *KRAS* gene [34].

In designing SuperSelective primers for assessing the abundance of these closely related mutations in multiplex assays, there are two principles that need to be kept in mind: (i) the foot sequence in each SuperSelective primer must not only distinguish against the wild-type sequence, it must also distinguish against each of the other closely related mutant sequences that can occur in the foot target site, so that the generation of amplicons by each SuperSelective primer only occurs when its intended mutant target is present in the sample; and (ii) in addition to not hybridizing to the intervening sequence in the target, the unique bridge sequence in each SuperSelective primer should not hybridize to the complements of the bridge sequences that are incorporated into the amplicons generated by the other SuperSelective primers.

The close localization of these mutations poses three potential problems that must be overcome if these mutations are to be simultaneously distinguished and quantitated in multiplex real-time PCR assays: (i) the sequences of the different amplicons are so similar to each other that differently colored fluorescent probes cannot readily distinguish the different amplicons; (ii) since the different SuperSelective primers share an identical (or virtually identical) anchor sequence, it is necessary to find a means to ensure that in subsequent rounds of synthesis, each amplicon is only copied by the correct SuperSelective primer; and most importantly, (iii) the generation of amplicons from a relatively abundant mutant sequence will suppress the generation of amplicons from a different, less abundant mutant sequence. This occurs because the different amplicons are so similar to each other, that once the concentration of amplicons generated from a relatively abundant mutant becomes so high that those (+) and (-) amplicons readily form double strands during the annealing phase of an amplification cycle, then the (+) and (-) amplicons generated from a less abundant mutant, though not yet sufficiently concentrated to form double strands with each other, will form double-stranded heteroduplexes with the amplicons generated from the more abundant mutant. As a consequence, the ability of the primers for the less abundant mutant to access their amplicons is reduced, and the exponential amplification of the less abundant mutant is compromised, resulting in the inability to obtain a meaningful Ct value for the less abundant mutant. These problems cannot be overcome by existing priming strategies. However, we have been able to address all of these challenges by virtue of alterations in the design of the SuperSelective primers, and by changing the concentrations of the primers used in multiplex assays.

### Addition of different identifying “tag” sequences to the 5' end of each SuperSelective primer

Since the entire sequence of each SuperSelective primer is incorporated into the (+) amplicons generated from that primer, we have added a unique identifying 32-nucleotide-long “tag” sequence to the 5' end of each SuperSelective primer used in multiplex PCR assays. Consequently, when these (+) amplicons are copied in subsequent rounds of synthesis, the resulting (-) amplicons contain a unique identifying complementary sequence at their 3' ends. We also include in the assay mixture a set of molecular beacon probes, each designed to hybridize to one of the identifying sequences at the 3' end of the (-) amplicons, and each is labeled with a differently colored fluorophore. Since non-hybridized molecular beacons are virtually non-fluorescent, and hybridized molecular beacons fluoresce brightly in their characteristic color [30, 31], the amount of each type of (-) amplicon present during the annealing phase of each amplification cycle is automatically measured by the spectrofluorometric thermal cycler in which the PCR assays are carried out.

### Ensuring that each amplicon is only copied by its “correct” SuperSelective primer

When a SuperSelective primer initiates the synthesis of a (+) amplicon on a DNA template present in the original sample, the entire sequence of the SuperSelective primer is incorporated into that amplicon, including the unique bridge sequence and the foot sequence. Consequently, in subsequent rounds of amplification, when conventional reverse primers initiate synthesis on those (+) amplicons, the resulting (-) amplicons contain the complement of the SuperSelective primer’s bridge sequence in place of the intervening sequence that was present in the original DNA template (see Fig 2). Moreover, in multiplex assays, during the annealing phase of subsequent rounds of amplification, the complement of the incorporated bridge sequence and foot sequence present in those (-) amplicons is recognized by the bridge sequence and the foot sequence at the 3' ends of the “correct” SuperSelective primers, which then initiate the synthesis of correct (+) amplicons. The “incorrect” primers present in the multiplex reaction mixture, on the other hand, possess an entirely different bridge sequence and a slightly different foot sequence at their 3' end, so their 3' ends cannot bind to incorrect amplicons, and they cannot initiate synthesis on those amplicons. Although it is likely that the entire sequence of the correct primer (which is quite long) will bind to its complementary amplicon as the reaction mixture is lowered to the annealing temperature before the relatively shorter anchor sequence of an incorrect primer or the relatively shorter probe sequence of a molecular beacon can bind to that amplicon in each thermal cycle, the design of the SuperSelective primers ensures that only the correct primer can initiate synthesis.

### Preventing the significant formation of heteroduplexes that obscure the Ct values of rare mutants

In multiplex real-time PCR assays, where different target sequences are closely related, as occurs for the measurement of the abundance of mutations that are located in the same or adjacent codons, the amplicons generated from a more abundant mutant can form heteroduplexes with complementary amplicons generated from a less abundant mutant, which results in the premature inhibition of the exponential amplification of the less abundant mutant, thereby obscuring the Ct value of the less abundant mutant. We have discovered that the use of non-symmetric primer concentrations [35, 36], in which the concentration of each SuperSelective primer is limited, enables the Ct value of each mutant target sequence to be separately determined.

Apparently, the concentration of each SuperSelective primer in the initial reaction mixture is so low when non-symmetric primer concentrations are used that even if all of the SuperSelective primers for a more abundant mutant are incorporated into (+) amplicons, there are never enough of these (+) amplicons present to significantly inhibit the exponential amplification of less abundant mutants. There are two additional factors that promote the exponential amplification of less abundant mutants: (i) the long length of the SuperSelective primers for less abundant mutants (approximately 80 nucleotides) enables them to compete well against the limited number of (+) amplicons from the more abundant mutant (approximately 100 to 140 nucleotides) for binding to the (-) amplicons of the less abundant mutant; and (ii) even if a heteroduplex does form, the unique bridge sequence of the (+) amplicon of the more abundant mutant is not complementary to the unique bridge sequence complement in the (-) amplicon of the less abundant mutant, so a bubble forms in the heteroduplex (composed of the different bridge sequences) which provides a single-stranded site to which the SuperSelective primer for the less abundant mutant can bind, and through strand displacement, initiate the synthesis of additional (+) amplicons of the less abundant mutant.

Moreover, after all of the SuperSelective primers for a given mutant are incorporated into (+) amplicons, the presence of the excess common conventional reverse primer enables complementary (-) amplicons to continue to be synthesized. During this latter “linear” phase of amplification, the distinctively colored molecular beacons that bind to those (-) amplicons face virtually no competition from the less abundant complementary (+) amplicons, and the time course of the growth of the brightly colored fluorescence signals from those amplicons provides a Ct value for each mutant that is inversely linearly proportional to the abundance of that mutant in the original sample.

### Fine-tuning SuperSelective primer sets for use in real-time multiplex PCR assays

The clinical goal of multiplex real-time PCR assays that utilize SuperSelective primers is to measure the abundance of different rare DNA fragments that possess mutations relevant to cancer, and to determine their abundance in relation to the amount of DNA present in the sample. However, in these multiplex assays each SuperSelective primer possesses a somewhat different foot sequence, which affects the strength of the foot hybrid (enthalpy), and each SuperSelective primer possesses a distinctly different bridge sequence, whose length and rigidity [37] as part of the bubble affects the probability that the foot hybrid will form (entropy). Consequently, the Ct value observed in a multiplex PCR assay for a given number of DNA fragments containing a particular mutation can occur somewhat earlier or somewhat later than the Ct values observed for the same number of DNA fragments containing a different mutation, thereby making it difficult to inter-compare the abundance of the different mutant fragments.

However, as demonstrated in the experiment whose results are shown in Fig 4B, relatively large increases in bubble circumference produce relatively small increases in the Ct values obtained with a SuperSelective primer. It is therefore possible to make small changes in the length of a SuperSelective primer’s bridge sequence in order to fine-tune the Ct value that will be obtained for a given number of target sequences (without significantly affecting the selectivity of the primer). It is even possible to fine-tune the resulting Ct value by making small changes in the nucleotide sequence of its bridge (without changing the number of nucleotides), thereby altering the rigidity of the bridge. In this manner, the design of each member of a set of SuperSelective primers that will be used in a multiplex PCR assay can be adjusted (based on preliminary experimentation) so that the Ct value that is obtained with each primer in a multiplex assay will accurately reflect the number of its target sequences that are present in a sample; and no matter which target sequence is amplified, a given Ct value will represent the same number of target fragments originally present in the sample. Consequently, the resulting set of Ct values for all of the different target sequences whose abundance is measured in the same assay will be directly inter-comparable.

**Fig 6:**
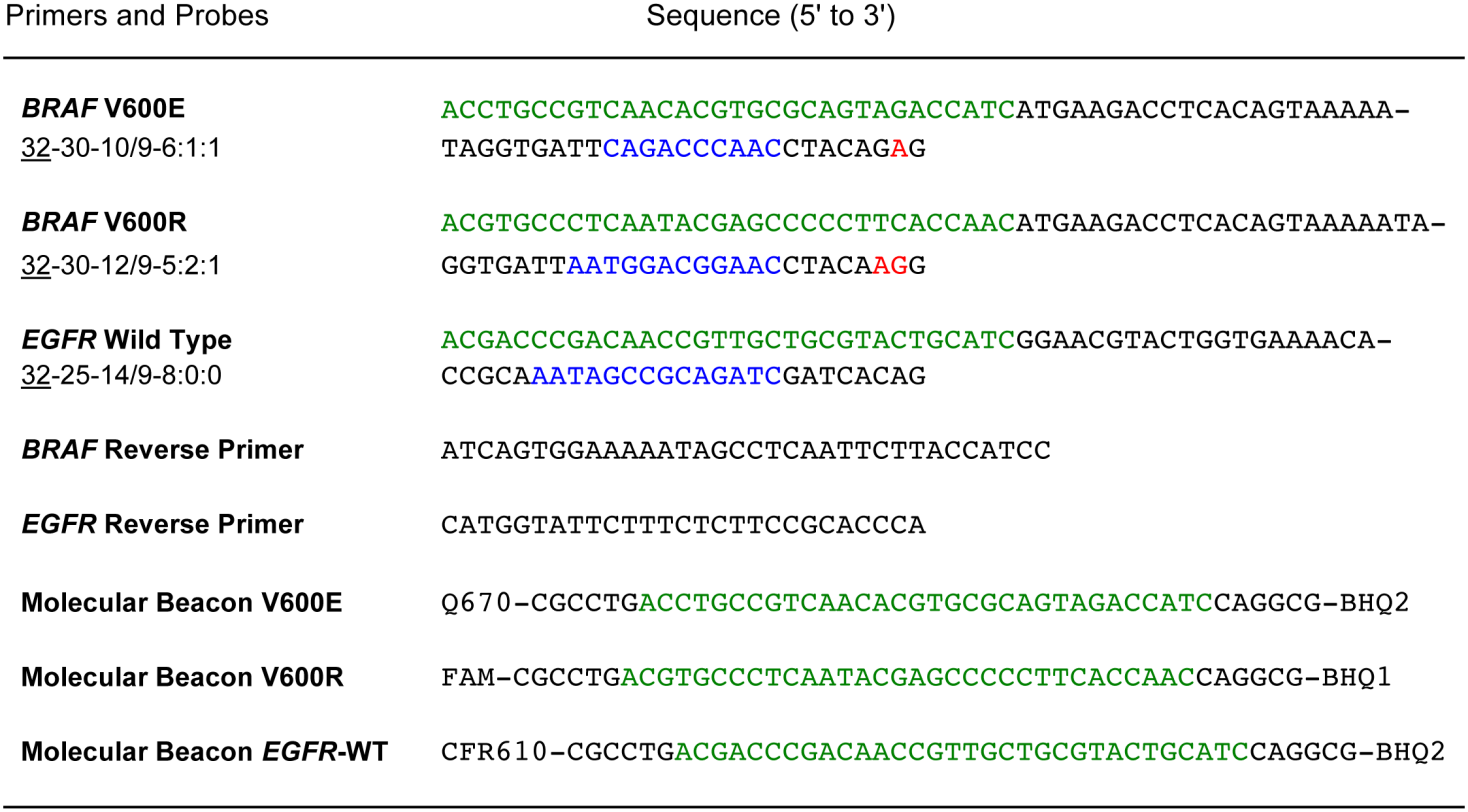
Fine-tuned primers and molecular beacons utilized in multiplex PCR assays. The *BRAF* SuperSelective primers whose sequences are shown here bind to the opposite strand to which the *BRAF* V600E SuperSelective primers shown in Fig 3 bind. The target sequence of the *BRAF* V600R SuperSelective primer mismatches the wild-type target sequence (and the *BRAF* V600E target sequence) at two adjacent nucleotides, so the *BRAF* V600R SuperSelective primer has two interrogating nucleotides. The *BRAF* target sequence is 136 base pairs long; and the *EGFR* target sequence is 90 base pairs long. The unique 32-nucleotide-long tag sequences at the 5' end of each SuperSelective primer is shown in green; the unique bridge sequence within each SuperSelective primer is shown in blue; and the interrogating nucleotides in the 3' foot sequences are shown in red. The complementary arm sequences of each molecular beacon are shown in black; and their probe sequences are shown in green. The differently colored fluorophores linked to the 5' ends of the molecular beacons are Quasar^®^ 670, fluorescein, and CAL Fluor^®^ Red 610; and the quencher moieties linked to the 3' ends of the molecular beacons are Black Hole Quencher^®^-1, and Black Hole Quencher^®^-2.

Fig 6 shows the sequences of a fine-tuned set of *BRAF* SuperSelective primers that we prepared for use in model multiplex assays. Each primer contained a unique bridge sequence. The bridge sequence of the *BRAF* V600R primer needed to be two nucleotides longer than the bridge sequences of the *BRA*F V600E primer in order to fine-tune the primer set. In addition, we prepared a fine-tuned SuperSelective primer for an unrelated *EGFR* wild-type sequence, whose Ct value serves as a reference for the amount of DNA in the sample. Each primer possessed a unique 32-nucleotide-long tag sequence at its 5' end (whose complement served as a target sequence for a uniquely colored molecular beacon probe).

### Use of non-symmetric primer concentrations in multiplex PCR assays

To demonstrate the advantage of using non-symmetric primer concentrations in multiplex assays, we prepared samples of restriction enzyme-digested plasmids that contained 10,000 copies of *BRAF* wild-type fragments (to simulate the abundant DNA fragments likely to be present in a liquid biopsy), 1,000 copies of *BRAF* V600E mutant target sequences, and differing amounts of *BRAF* V600R mutant target sequences (10; 39; 156; 625; 2,500; or 10,000 copies), and carried out two sets of multiplex real-time PCR assays. One set was run with symmetric primer concentrations (500 nM fine-tuned SuperSelective primer *BRAF* V600E 32-30-10/9-6:1:1; 500 nM fine-tuned SuperSelective primer *BRAF* V600R 32-30-12/9-5:2:1; and 1,000 nM conventional *BRAF* common reverse primer); and the other set was run with non-symmetric primer concentrations (60 nM fine-tuned SuperSelective primer *BRAF* V600E 32-30-10/9-6:1:1; 60 nM SuperSelective fine-tuned primer *BRAF* V600R 32-30-12/9-5:2:1; and 1,000 nM conventional *BRAF* common reverse primer). All of the reactions contained 300 nM of the Quasar^®^ 670-labeled *BRAF* V600E-specific molecular beacon and 300 nM of the fluorescein-labeled *BRAF* V600R-specific molecular beacon. Otherwise identical control reactions containing no target molecules were also carried out with each set of samples, to confirm that these relatively long primers (which include 32-nucleotide tag sequences at their 5' ends) do not generate false amplicons. The computer program controlling the thermal cycler in which the assays took place separately recorded the intensity generated by each of the two differently colored molecular beacons during the annealing stage of each thermal cycle.

**Fig 7:**
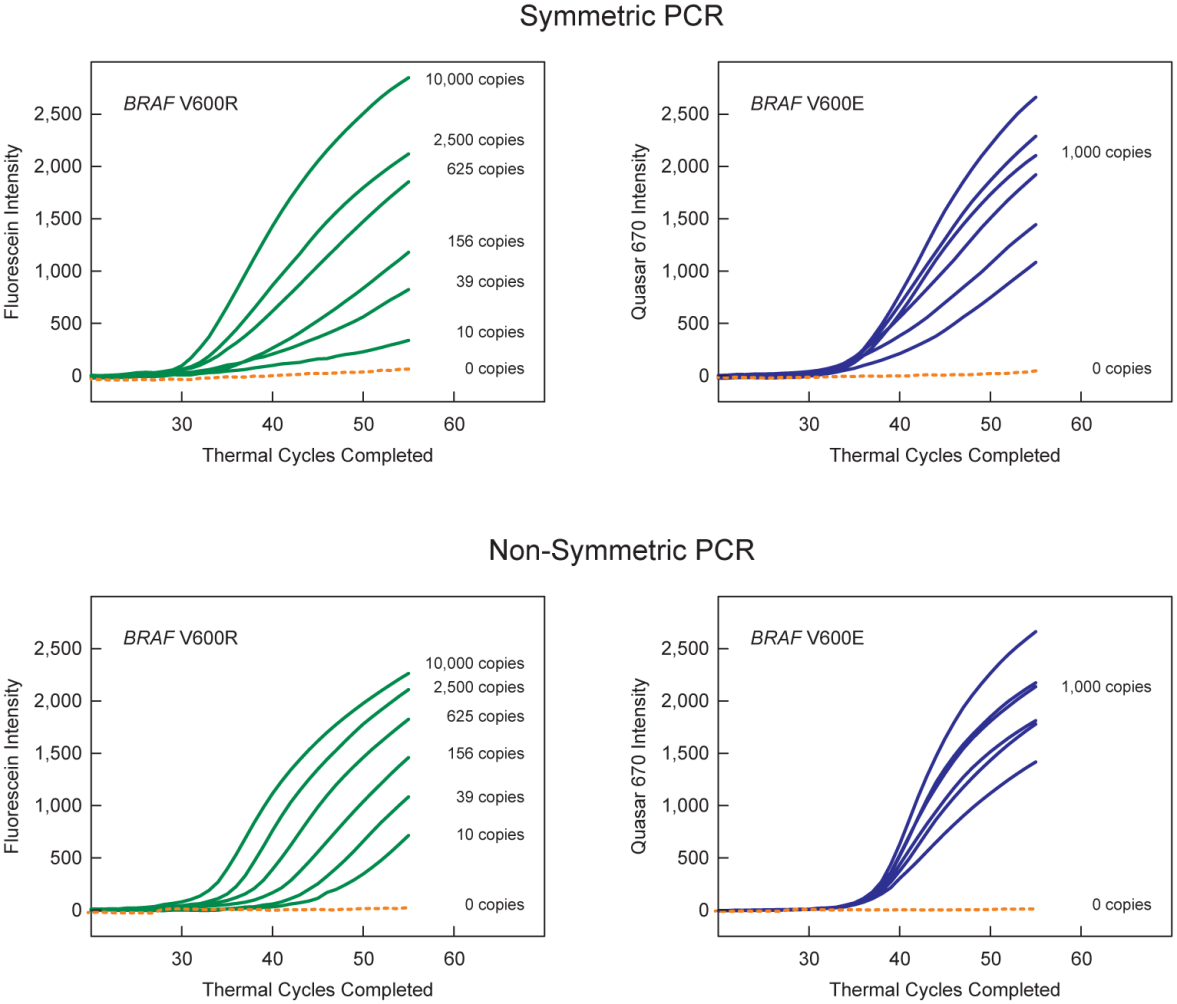
Demonstration of the advantage of using non-symmetric primer concentrations (as opposed to using symmetric primer concentrations) in multiplex PCR assays that quantitate different mutations that, although occurring in different cells, are located in the same codon. These assays contained fine-tuned SuperSelective primers *BRAF* V600E 32-30-10/9-6:1:1 and *BRAF* V600R 32-30-12/9-5:2:1 (where the underlined value reflects the length of the 5'-tag sequence), and two differently colored molecular beacon probes to detect the resulting amplicons. The left-hand panels show the results from the fluorescein-labeled *BRAF* V600R-specific molecular beacons (green curves); and the right-hand panels show the results from the Quasar^®^ 670-labeled *BRAF* V600E-specific molecular beacons (blue curves). Samples without any templates were also run as controls (dotted orange curves). The symmetric PCR reactions, whose results are shown in the upper panels, contained 500 nM of each SuperSelective primer and 1,000 nM of the conventional *BRAF* common reverse primer. The non-symmetric PCR reactions, whose results are shown in the lower panels, contained only 60 nM of each SuperSelective primer and 1,000 nM of the conventional *BRAF* common reverse primer.

The results (shown in Fig 7) demonstrate that in multiplex assays under symmetric PCR conditions, amplicon strands generated from less abundant *BRAF* V600R target sequences form heteroduplexes with complementary amplicon strands generated from more abundant *BRAF* V600E target sequences, resulting in the threshold values of the less abundant *BRAF* V600R target sequences being obscured by virtue of their binding to the more abundant *BRAF* V600E amplicons. On the other hand, under non-symmetric PCR conditions, heteroduplexes rarely form, thereby enabling the independent quantitation of the relative abundance of the *BRAF* V600R target sequences present in each sample. These results demonstrate that the Ct values determined in multiplex real-time PCR assays containing non-symmetric primer concentrations provide quantitative results, despite simultaneously detecting different mutations that occur in the same codon.

### Multiplex PCR assays that also amplify an unrelated reference wild-type sequence

The unique ability to choose the length and nucleotide sequence for the bridge of each SuperSelective primer present in real-time multiplex assays enables the inclusion of a fine-tuned SuperSelective primer and a conventional reverse primer that are specific for the amplification of a reference wild-type sequence present in the sample that is unrelated to the rare mutations of interest. The generation of amplicons from this reference sequence (reflected by the fluorescence of a distinctively colored molecular beacon) serves as an internal control to ensure that the PCR assay is functioning well. Moreover, the Ct value of these wild-type amplicons reflects the amount of DNA present in the sample, and if that Ct value turns out to be higher than a pre-determined value, the assay results would be ignored due to there being too little DNA in the sample for the rare target mutations, if they exist, to be present. But most significantly, the use of a fine-tuned SuperSelective primer that generates a Ct value that reflects the amount of DNA in the sample enables the Ct values generated by the similarly fine-tuned SuperSelective primers for the mutant target sequences to be directly inter-compared. The difference between the Ct value of a rare mutant and the Ct value of the reference gene is a direct reflection of their relative abundance, and this comparison does not require a pre-determination of the amount of DNA in the sample.

In choosing the reference gene segment to amplify in multiplex assays, we prefer to not use the wild-type sequence of the related mutant sequences whose abundances are being measured, because the resulting abundant wild-type amplicons could form heteroduplexes with the mutant amplicons, potentially interfering with exponential amplification of those mutant amplicons. Moreover, we prefer to not use a reference gene whose copy number may be amplified in a patient’s cancerous cells, as this could distort the results.

In order to demonstrate the value of including primers for a reference wild-type gene in non-symmetric multiplex assays employing fine-tuned SuperSelective primers, we carried out model triplex real-time PCR assays that simultaneously amplified *BRAF* V600E mutant sequences, *BRAF* V600R mutant sequences, and a reference wild-type sequence (*EGFR*). Each reaction contained five primers (60 nM SuperSelective primer *BRAF* V600E 32-30-10/9-6:1:1, 60 nM SuperSelective primer *BRAF* V600R 32-30-12/9-5:2:1, 1,000 nM conventional *BRAF* common reverse primer, 60 nM SuperSelective primer *EGFR* wild-type 32-25-14/9-8:0:0, and 500 nM conventional *EGFR* reverse primer) and three differently colored molecular beacon probes to detect the resulting amplicons. The sequences of all these oligonucleotides are shown in Fig 6. Two sets of reactions were carried out. The first set contained 10,000 *EGFR* wild-type fragments, 1,000 *BRAF* V600R fragments, and different quantities of *BRAF* V600E fragments (0; 10, 39; 156; 625; and 2,500). The second set contained 10,000 *EGFR* wild-type fragments, 1,000 *BRAF* V600E fragments, and different quantities of *BRAF* V600R fragments (0; 10, 39; 156; 625; and 2,500). Both sets also contained 10,000 *BRAF* wild-type fragments to simulate actual samples, though the reactions did not include a SuperSelective primer for the exponential amplification of the *BRAF* wild-type sequence.

**Fig 8:**
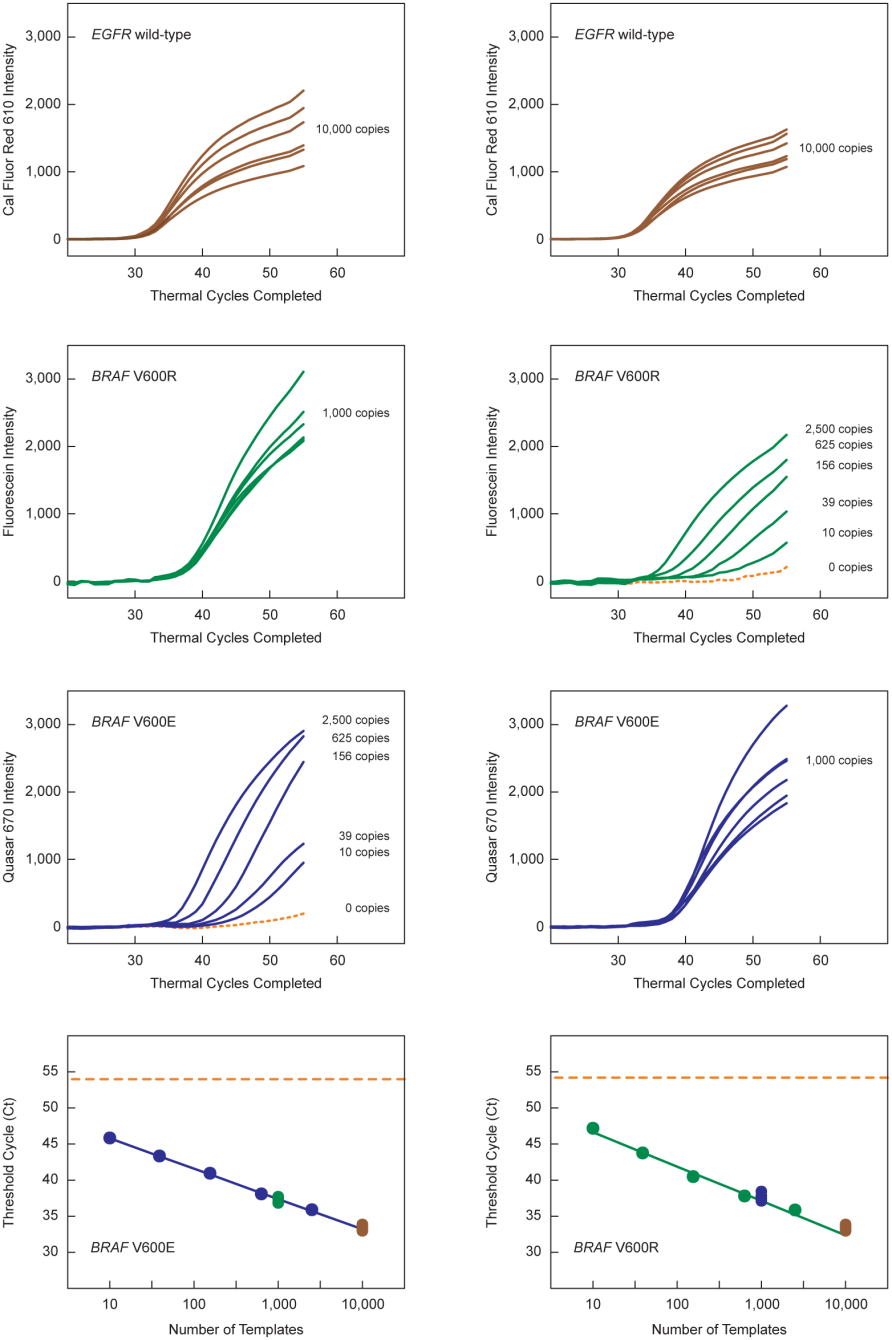
Demonstration of the value of including primers for a reference wild-type gene in non-symmetric multiplex PCR assays employing fine-tuned SuperSelective primers. The four left-hand panels show the results obtained for reactions containing 10,000 *EGFR* wild-type fragments, 10,000 *BRAF* wild-type fragments, 1,000 *BRAF* V600R fragments, and different quantities of *BRAF* V600E fragments (0; 10, 39; 156; 625; and 2,500 copies). The four right-hand panels show the results obtained for reactions containing 10,000 *EGFR* wild-type fragments, 10,000 *BRAF* wild-type fragments, 1,000 *BRAF* V600E fragments, and different quantities of *BRAF* V600R fragments (0; 10, 39; 156; 625; and 2,500 copies). The panels in the top row show the results recorded by the CAL Fluor^®^ Red 610 *EGFR* wild-type-specific molecular beacon; the panels in the second row show the results recorded by the fluorescein-labeled *BRAF* V600R-specific molecular beacon; and the panels in the third row show the results recorded by the Quasar^®^ 670-labeled *BRAF* V600E-specific molecular beacon. The lower panels plot the logarithm of the number of variable *BRAF* target fragments present in each reaction against the Ct value that was obtained. The Ct values for the 1,000 fragments of the non-variable *BRAF* mutant in each sample, as well as the Ct values for the 10,000 *EGFR* reference wild-type fragments present in each sample, are also plotted on the same line. The dotted orange lines indicate the Ct value of the sample containing no *BRAF* V600E mutant targets (left-hand panel) and no *BRAF* V600R targets (right-hand panel).

The results (shown in Fig 8) demonstrate that the Ct values determined for each target sequence, when plotted against the logarithm of the number of those target sequences present in the sample, all lie on a straight line, confirming that the designs of the SuperSelective primers used in these multiplex assays were fine-tuned so that the different Ct values could be directly inter-compared. Consequently, the difference (ΔCt) between the Ct value obtained for each mutant target sequences in the sample and the Ct value obtained for the reference wild-type target sequence reflects the abundance of those mutant target sequences relative to the abundance of the reference wild-type target sequence, irrespective of the amount of DNA present in the sample. It is probable that the amount of DNA in a liquid biopsy sample taken from an individual will vary considerably in the course of a day. However, the abundance of each target mutation relative to the abundance of a reference gene is likely to be a reliable indicator of the underlying clinical situation.

## Discussion

A key feature of SuperSelective primer design is that the foot sequences form unusually short hybrids with the template molecules present in the original sample. Consequently, mismatched wild-type hybrids are considerably weaker than perfectly complementary mutant hybrids. Under the annealing conditions that we used in our experiments, the thermodynamic consequences of this difference in hybrid stability is that, at any given moment, mismatched wild-type hybrids are much less likely to be present than perfectly complementary mutant hybrids; and amplicons are therefore less likely to be generated on wild-type templates than on mutant templates. This view is confirmed by the observation that discrimination increases as foot length is decreased (see Fig 4A). Moreover, the A-T rich 5:1:1 foot sequence of the *BRAF* V600E primer, which forms relatively weak hybrids, was more discriminatory than the G-C rich 5:1:1 foot sequence of the *EGFR* L858R primer, which forms stronger hybrids.

### Thermodynamic basis of selectivity

However, thermodynamic differences in the equilibrium abundance of the perfectly complementary foot hybrids formed with the mutant, compared to the lower equilibrium abundance of the weaker mismatched foot hybrids formed with the wild type, are insufficient to explain the significant differences that were observed in the probability of generating mutant amplicons, compared to the probability of generating wild-type amplicons. For example, the Ct value of the reaction containing only 10^6^ *BRAF* wild-type fragments occurred more than 20 cycles later than the Ct value of the reaction containing 10^6^ *BRAF* V600E fragments in the presence of 10^6^ *BRAF* wild-type fragments (see Fig 1C). If classical thermodynamic parameters were the only factors affecting selectivity, the equilibrium abundance of the mutant foot hybrids would have needed to be of the order of 1,000,000-fold greater (i.e., 2^20^) than the equilibrium abundance of the wild-type foot hybrids to account for a 20-cycle difference in the observed threshold values, and this great difference is highly unlikely to arise solely from thermodynamic considerations.

### Possible enzymatic basis of enhanced selectivity

We therefore considered whether or not the presence or absence of a 3'-terminal base pair formed by the foot sequence affects the initiation of amplicon synthesis, which is the enzymatic discriminatory principle of ARMS primers [9]. We utilized two SuperSelective primers: (i) one primer possessed a 5:1:1 foot sequence, whose interrogating nucleotide was located at the penultimate position from the primer’s 3' end, so that its two 3'-terminal nucleotides were unlikely to form base pairs with the wild-type target sequence under the 60°C annealing conditions; and (ii) the other primer was identical, except that it possessed a 5:0:0 foot sequence (i.e., its foot was only five nucleotides in length, lacking the two 3'-terminal nucleotides of the other primer) and was therefore perfectly complementary to both the mutant target sequence and the wild-type target sequence. The sequences of these two *EGFR* L858R primers (24-14/14-5:1:1 and 24-14/14-5:0:0) are shown in Fig 3. Two monoplex PCR assays were carried out with each of the primers, one initiated with 1,000,000 copies of mutant template, and one initiated with 1,000,000 copies of wild-type template. The threshold cycles that were observed are listed in Table 3. The results show that the 40.7 Ct value obtained for the reaction containing wild-type templates and the primer with the 5:1:1 foot sequence (which forms a five-nucleotide-long hybrid in which the two additional nucleotides at its 3' end do not form base pairs) was virtually identical to the 39.4 Ct value obtained for the reaction containing wild-type templates and the truncated primer with the 5:0:0 foot sequence (which also forms a five-nucleotide-long hybrid, but does not possess two unpaired 3'-terminal nucleotides).

**Table 3:** Comparison of Ct values obtained with SuperSelective *EGFR* L858R primers that can form mismatched 3'-terminal base pairs to the Ct values obtained with corresponding primers that cannot form 3'-terminal base pairs.

	Threshold Cycle (Ct)		
Primer	10^6^ Mutant Templates	10^6^ Wild-type Templates	ΔCt
24-14/14-5:1:1	23.1	40.7	17.6
24-14/14-5:0:0	39.7	39.4	0.3

This result demonstrates that enzymatic discrimination against unpaired 3'-terminal nucleotides does not play a significant role in the extraordinary selectivity of SuperSelective primers. Had there been significant enzymatic discrimination (due to ARMS) as a consequence of there being unpaired 3'-terminal nucleotides at the end of the hybrids formed by the 5:1:1 foot with the wild-type templates, then the Ct value of that hybrid would have occurred significantly later than the same-length completely complementary hybrids formed by the truncated 5:0:0 foot with the wild-type templates. This result suggests that there is a non-enzymatic basis for the extraordinary discrimination that occurs when the foot hybrid formed by SuperSelective primers contains mismatched base pairs.

### Kinetic basis of enhanced selectivity

We believe that there is another discriminatory factor that arises from the short length of the weak hybrids formed by a SuperSelective primer’s foot sequence. Once a foot hybrid forms, it must interact with a DNA polymerase molecule in such a manner that it can form a stable complex that leads to the generation of an amplicon. Under annealing conditions, short hybrids, only a few base pairs in length, have melting temperatures far below the 60°C annealing temperature, and their mean persistence times are measured in milliseconds [38, 39], as opposed to the 24-base pair anchor hybrids, whose mean persistence time is measured in minutes. Since mismatched wild-type foot hybrids are shorter and weaker than perfectly complementary mutant foot hybrids, the mean persistence times of wild-type foot hybrids are significantly shorter than the mean persistence time of mutant foot hybrids. Consequently, as the hybrids and the DNA polymerase molecules move about chaotically under annealing conditions, there is a much lower probability that a very short-lived (mismatched) wild-type foot hybrid will encounter a DNA polymerase molecule before that hybrid dissociates, than the probability that a longer-lived (perfectly complementary) mutant foot hybrid will encounter a DNA polymerase molecule before it dissociates. Thus, the kinetic factors that arise from the different mean persistence times of the foot hybrids add to the thermodynamic factors that affect differences in hybrid abundance, and the result is the extraordinary selectivity of SuperSelective primers. Consequently, hybrids formed with the mismatched foot sequences of SuperSelective primers persist for such a short period of time under annealing conditions that they virtually all fail to encounter and form an active complex with a DNA polymerase molecule before they dissociate. A kinetic basis for the enhanced selectivity of SuperSelective primers, as opposed to an enzymatic (ARMS) basis for their enhanced selectivity, is supported by the observation that the Ct value obtained with a truncated SuperSelective primer (5:0:0 foot) and 1,000,000 wild-type targets is virtually identical to the Ct value obtained with the corresponding full-length SuperSelective primer (5:1:1 foot) and 1,000,000 wild-type targets (Table 3).

### Role of the bubble formed by a SuperSelective primer and its target

Another consideration in determining the basis of discrimination is the observation that the greater the circumference of the bubble formed by the hybridization of a SuperSelective primer to an original template molecule, the greater is the suppression of wild-type amplicon synthesis relative to the suppression of mutant amplicon synthesis (see Fig 4B). From a thermodynamic point of view, larger bubbles should reduce the equilibrium abundance of both the wild-type hybrids and the mutant hybrids, but should not alter their relative abundance [40]. However, from a kinetic point of view, it is appropriate to consider the forces that impinge upon the single-stranded bubble that connects the foot hybrid to the anchor hybrid, because the bubble sequences are subject to perturbations from random Brownian motions of the water molecules in the reaction mixture that create a force with the potential to pull the foot hybrids apart. The greater the circumference of the bubble, the greater is this potentially disruptive force. Significantly, mismatched wild-type hybrids, which are weaker than perfectly complementary mutant hybrids, are more easily pulled apart. Thus, mismatched wild-type hybrids not only exist for a shorter length of time due to their lower stability, they are also more easily pulled apart by the random forces that impinge upon the bubble [29], further reducing their mean persistence time, and consequently increasing the selectivity of the assay.

### Future directions

Utilizing the design principles explored in this report, real-time PCR assays can be developed that simultaneously assess the abundance of a number of different clinically relevant rare mutations in plasma samples. These assays will contain sets of fine-tuned SuperSelective primers and uniquely colored detector probes that enable the abundance of each target mutation to be compared to the abundance of a single reference wild-type sequence amplified in the same assay, simply by observing the difference between the Ct value of each target mutation (if it is present in the sample) and the Ct value of the reference wild-type sequence.

In addition to measuring the abundance of closely related mutations that arise in the same codon, the abundance of mutations located in other genes can simultaneously be measured, the only limit being the number of differently colored fluorescence signals that can be distinguished by the spectrofluorometric thermal cycler in which the assays are carried out. The sequences of the conventional reverse primers chosen for these assays should be designed to bind close to the mutation of interest, maximizing the ability to generate amplicons from the short DNA fragments present in plasma [41]. In addition to plasma samples, these assays may prove useful for the analysis of other accessible clinical samples, such as saliva, urine, lymph, and stool.

The ultimate value of these PCR assays will need to be determined in carefully controlled clinical studies that ascertain how the results of the assays can be utilized to enable adjustments in therapy that significantly prolong the life (and quality of life) of patients with different cancers. Current techniques for analyzing liquid biopsies, such as DNA sequence analysis [42] and digital PCR [43], are complex, costly, and have limited sensitivity [44]. Hopefully, real-time PCR assays that utilize SuperSelective primers will provide a low-cost, extremely sensitive alternative that can be carried out in virtually any of the many spectrofluorometric thermal cyclers available in hospitals and laboratories around the world.
